# Male Breast Cancer Complicated With Leukocytosis Resembling Leukemia Reaction After Chemotherapy: A Case Report

**DOI:** 10.1002/cnr2.70280

**Published:** 2025-07-22

**Authors:** Yanze Liu, Jiaqi Liu

**Affiliations:** ^1^ Department of Breast and Thyroid Surgery Zibo Central Hospital Zibo Shandong China

**Keywords:** chemotherapy, leukocytosis resembling leukemia reaction, male breast cancer

## Abstract

**Introduction:**

Male breast cancer (MBC) accounts for less than 1% of all cancers in men, with invasive ductal carcinoma being the most common type. The chemotherapy regimens used for MBC are similar to those for female breast cancer. However, the incidence of chemotherapy‐induced complications such as leukocytosis resembling leukemia reaction is not well documented in MBC. This case highlights a rare complication in an MBC patient, induced by prophylactic PEG‐rhG‐CSF following chemotherapy.

**Case Presentation:**

A 51‐year‐old male with left breast invasive ductal carcinoma underwent modified radical mastectomy. Postoperative pathology revealed high‐risk features, and the patient received 8 cycles of chemotherapy with the ddAC‐T regimen, followed by PEG‐rhG‐CSF for febrile neutropenia prevention. After the fifth chemotherapy cycle, the patient developed leukocytosis resembling leukemia reaction, characterized by a white blood cell count exceeding 50 × 10^9^/L, along with intermittent fever up to 42.5°C. The condition was attributed to the PEG‐rhG‐CSF administration, and the patient was treated with NSAIDs and dexamethasone. Leukocytosis resolved after adjusting the PEG‐rhG‐CSF dose.

**Conclusion:**

Leukocytosis resembling leukemia reaction induced by PEG‐rhG‐CSF post‐chemotherapy is a rare complication, particularly in MBC patients. This case underscores the importance of careful monitoring and differential diagnosis to avoid misdiagnosis and unnecessary interventions. Personalized treatment strategies and dose adjustments for PEG‐rhG‐CSF are crucial in managing this rare reaction, emphasizing the need for awareness and individualized care in MBC patients undergoing chemotherapy.

## 1 Introduction

Male breast cancer (MBC) accounts for less than 1% of all cancers in men and less than 1% of breast cancers (BCs) overall [1]. The most common pathological type of MBC is invasive ductal carcinoma (IDC) [2]. The chemotherapy drugs and regimens used for MBC are similar to those used for female BC. The NCCN guidelines for the standardized management of chemotherapy‐induced neutropenia (CIN) recommend the primary prophylactic use of G‐CSF for high‐risk febrile neutropenia (FN) chemotherapy regimens [3]. These guidelines also mention rare adverse reactions, such as leukocytosis resembling leukemia reaction [4]. Leukocytosis resembling leukemia reaction induced by long‐acting PEG‐rhG‐CSF post‐chemotherapy is rare in MBC. Herein, we report a unique case of MBC complicated by a leukocytosis resembling leukemia reaction caused by PEG‐rhG‐CSF after chemotherapy. This case report has undergone ethics committee review by the Zibo Central Hospital (ethical approval number: 2025 Research No. 41) and has obtained informed consent from the patient.

## 2 Clinical Case

**FIGURE 1:**
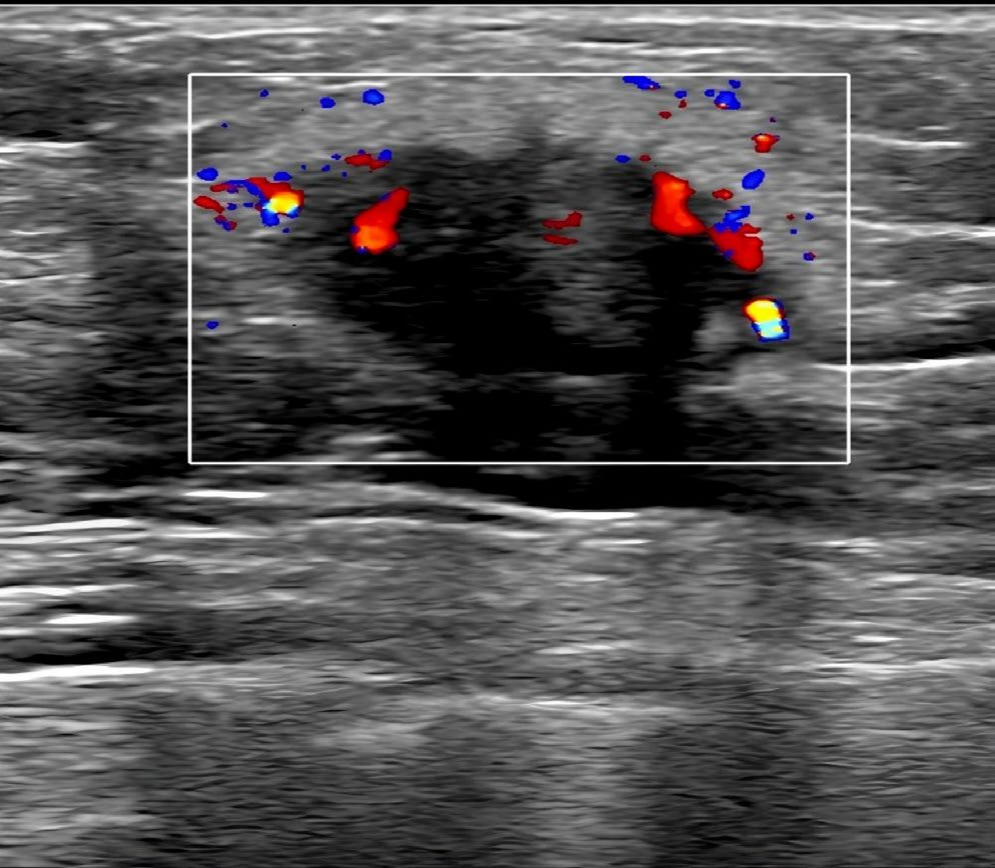
Breast ultrasonography showing a hypoechoic nodule measuring 13 × 13 × 8 mm with clear boundaries, irregular shape, and inhomogeneous internal echo located beneath the left nipple.

A 51‐year‐old male presented with a left breast lump that had been detected for 1 year. He was admitted to Zibo Central Hospital on November 27, 2022. Ultrasonography of the breast and axillary lymph nodes revealed a hypoechoic nodule measuring 13 × 13 × 8 mm with a clear boundary, irregular shape, and inhomogeneous internal echo beneath the left nipple. Color Doppler flow imaging (CDFI) detected a blood flow signal. Multiple enlarged lymph nodes were detected in the left sub‐axilla, with the largest measuring 13 × 6 mm, having clear boundaries, a distinct cortex and medulla structure with inhomogeneous internal echo, and CDFI also detected a blood flow signal (Figure 1). These findings indicated a high risk of malignancy (BIRADS‐4b). The chest CT scan showed no tumor metastasis. Subsequently, the patient underwent a modified radical mastectomy for left BC at Zibo Central Hospital on December 2, 2022.

Postoperative pathology reported grade III left breast IDC with focal interstitial lymphocyte infiltration (tumor volume: 1.5 × 1.5 × 0.8 cm), and a tumor thrombus was found in the vessel. Metastasis was detected in 1 of 3 left sentinel lymph nodes, but no metastasis was found in 7 left axillary lymph nodes. There was no tumor invasion in the interstitial nerve fiber bundle, nipple, and subpapillary tissue, and no cancer cells were found in the deep and peripheral tangents. Immunohistochemistry showed the following results: CKD5/6 was partially positive, P63 was negative, Ki‐67 was positive (*S* = 20%), estrogen receptor (ER) was strongly positive (*S* = 80%), progesterone receptor (PR) was moderately positive (*S* = 25%), androgen receptor (AR) was moderately positive (*S* = 50%), and human epidermal growth factor receptor 2 (HER2) was moderately positive. A subsequent fluorescence in situ hybridization (FISH) test indicated that HER2 was negative.

**FIGURE 2:**
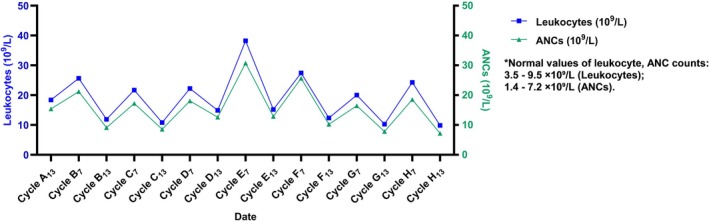
Changes in leukocytes and ANCs over 8 chemotherapy cycles. ANC, absolute neutrophil count. (The 50‐year‐old male patient received the AC‐T chemotherapy regimen for 8 cycles [Cycle A to Cycle H]. He was instructed to receive a subcutaneous injection of Mecapegfilgrastim [a long‐acting PEG‐rhG‐CSF] 6 mg within 48 h after chemotherapy. He rechecked his hematological analysis on the 7th and 13th days after each chemotherapy session to monitor changes in leukocytes and ANCs over 8 chemotherapy cycles). ANC, absolute neutrophil count. *Normal values of leukocyte, ANC counts: 3.5–9.5 × 10^9^/L (Leukocytes); 1.4–7.2 × 10^9^/L (ANCs).

The patient had a height of 181 cm and a weight of 70 kg, resulting in a body surface area of approximately 1.89 m^2^. He was prescribed doxorubicin (35 mg/m^2^), cyclophosphamide (600 mg/m^2^), and albumin‐bound paclitaxel (260 mg/m^2^) for 8 cycles, followed by radiotherapy and tamoxifen for 10 years. He agreed to this regimen. The patient was instructed to receive a subcutaneous injection of Mecapegfilgrastim (a long‐acting PEG‐rhG‐CSF) 6 mg within 48 h after chemotherapy. He was also asked to recheck his hematological analysis on the 7th and 13th days after each chemotherapy session to monitor changes in leukocytes and absolute neutrophil counts (ANCs) over 8 chemotherapy cycles (Figure 2).

**FIGURE 3:**
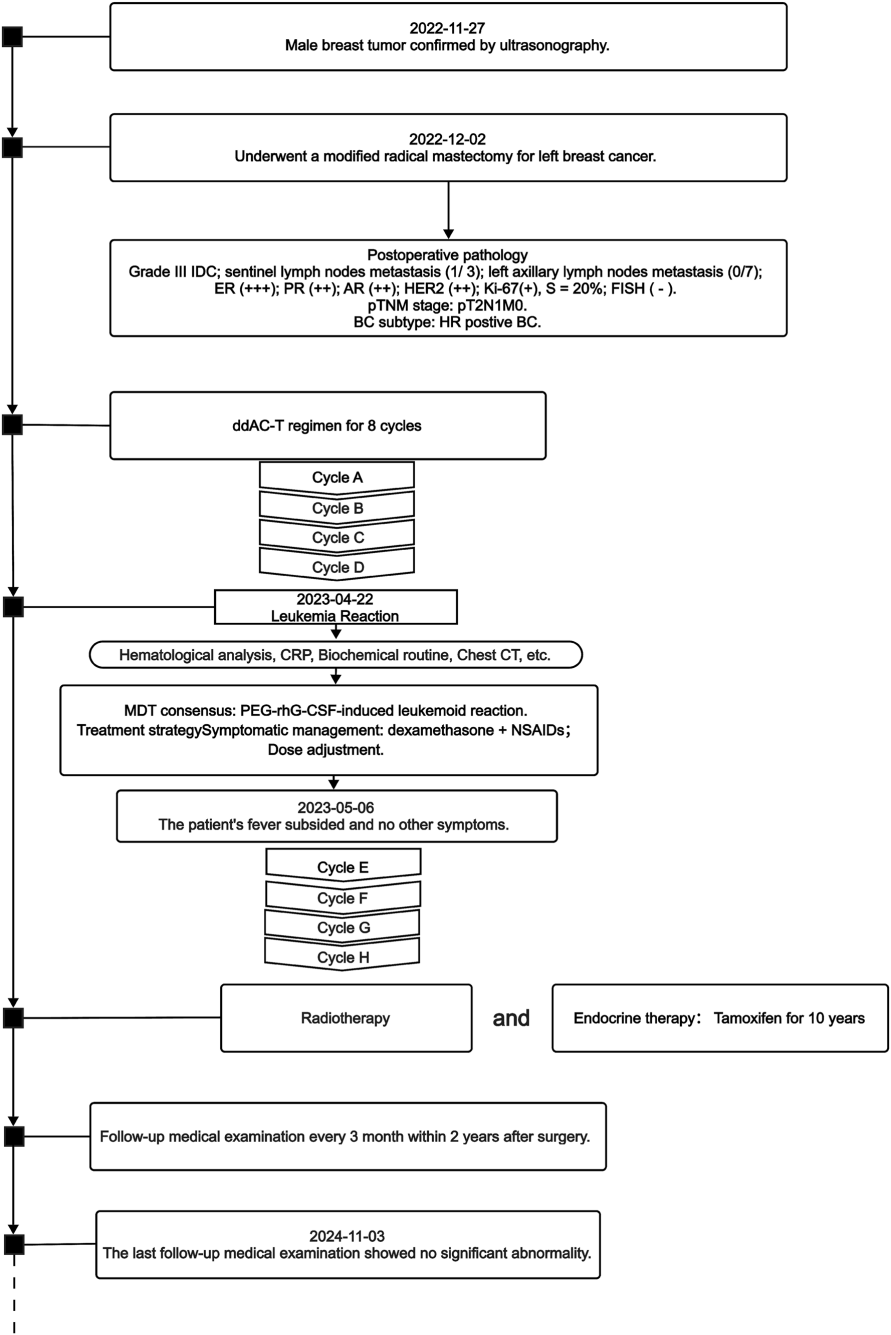
The progress of the patient's treatment. AR, androgen receptor; BC, breast cancer; CRP, C‐reactive protein; ER, estrogen receptor; FISH, fluorescence in situ hybridization; HER2, human epidermal growth factor receptor 2; HR, hormone receptor; MDT, multidisciplinary treatment; NSAIDs, nonsteroidal antiinflammatory drugs; PR, progesterone receptor. ddAC‐T: dd, Dose‐Dense (q2w); A, Doxorubicin; C, Cyclophosphamide; T, Paclitaxel.

The first four chemotherapy cycles progressed smoothly without adverse reactions such as fever. The average ANCs on the 7th and 13th days after the first four cycles were 18.82 × 10^9^/L and 11.42 × 10^9^/L, respectively. However, on the 5th day after the fifth chemotherapy cycle (April 22, 2023), the patient developed an intermittent fever, peaking at 39°C. Hematological analysis showed leukocytes at 38.24 × 10^9^/L and ANCs at 30.41 × 10^9^/L. He was treated with nonsteroidal anti‐inflammatory drugs (NSAIDs) for symptomatic relief. Despite this, the fever persisted, with the highest temperature reaching 42.5°C on April 23, 2023. The progress of the patient's treatment is illustrated in Figure 3.

**FIGURE 4:**
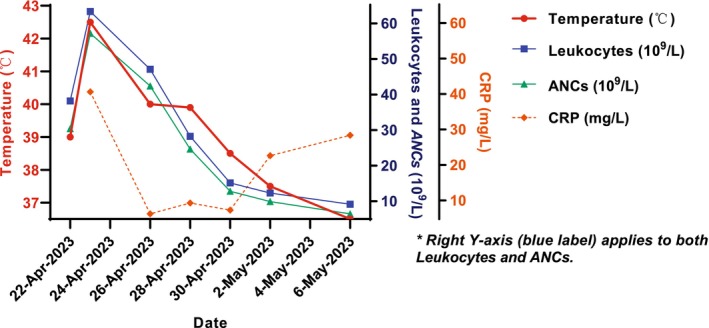
Changes in temperature, leukocytes, ANCs, and CRP following the fifth chemotherapy cycle. ANC, absolute neutrophil count; CRP, C‐reactive protein. Color conventions follow standard hematologic parameter representations in clinical studies. *Normal values of leukocyte, ANC, CRP counts: 3.5–9.5 × 10^9^/L (Leukocytes); 1.4–7.2 × 10^9^/L (ANCs); 0–5.00 mg/L (CRP).

Comprehensive examinations including hematological analysis, C‐reactive protein, biochemical routine, chest CT, urine routine, and fecal routine were conducted. Hematological analysis revealed leukocytes at 63.37 × 10^9^/L and ANCs at 57.22 × 10^9^/L, with C‐reactive protein levels at 40.78 mg/L (Figure 4). Other tests showed no abnormalities. Consultations with the infection and hematology departments led to recommendations for additional tests: procalcitonin, interleukin‐6, β‐D‐glucan, serological examination for aspergillus, and morphological examination of abnormal leukocytes. The latter indicated that granulocytes accounted for 15%, with no atypical cells detected. Other tests also showed no abnormalities.

This case was discussed with the Multidisciplinary Team (MDT) for BC at Zibo Central Hospital. The MDT included specialists from the following departments: Breast Surgery, Oncology, Pathology, Radiology, Traditional Chinese Medicine, and Hematology. The team reviewed the patient's condition, particularly the rare leukocytosis resembling a leukemia reaction induced by PEG‐rhG‐CSF, and collectively recommended the appropriate treatment strategy, including symptomatic management and dose adjustments. This collaborative discussion ensured a comprehensive approach to the patient's treatment.

After discussions, it was unanimously considered that the leukocytosis resembling leukemia reaction was caused by PEG‐rhG‐CSF. The patient was treated with daily dexamethasone and NSAIDs for antipyretic therapy. Subsequent hematological analysis and C‐reactive protein tests showed the following results (Figure 4).

The changes in leukocytes and ANCs are illustrated in Figure 4. During this period, the patient continued to experience intermittent fever, with the highest temperatures recorded as follows: 40°C (April 26), 39.9°C (April 28), 38.5°C (April 30), and 37.5°C (May 2). The patient's fever subsided by May 6, 2023, and there were no other symptoms, allowing him to proceed with the sixth chemotherapy session. PEG‐rhG‐CSF (3 mg) was administered subcutaneously 24 h after chemotherapy. The last three rounds of chemotherapy were completed successfully without any adverse reactions such as fever. The average ANCs on the 7th day after each chemotherapy cycle were 14.07 × 10^9^/L, and on the 13th day after the last three cycles, the average ANCs were 8.35 × 10^9^/L. After completing chemotherapy, the patient subsequently underwent radiotherapy and initiated oral tamoxifen therapy 2 weeks post‐chemotherapy, with tamoxifen treatment spanning from June 17, 2023 to June 17, 2033.

The patient currently undergoes regular systemic examinations every 6 months, including breast tumor marker assays, chest CT, breast and axillary color Doppler ultrasonography, abdominal (hepatobiliary, pancreatic, and splenic) ultrasonography, cranial magnetic resonance imaging (MRI), and annual bone scans, with no evidence of disease recurrence detected. The patient had a follow‐up visit on November 3, 2024, at the outpatient clinic. All examinations, including hematological analysis, were within the normal range, and no signs of recurrence or metastasis were observed. The patient is currently in stable condition and continues to be monitored every 3 months within 2 years after surgery (August 6, 2023; November 5, 2023; February 4, 2024; May 5, 2024; August 4, 2024; November 3, 2024) (Figure 3). Postoperative follow‐up medical examinations were conducted every 6 months after 2 years. The patient has not attended the follow‐up visit as of (November 3, 2024) due to personal reasons.

## 3 Discussion

The incidence of MBC appears to be slowly rising in recent years [2]. Compared with female BC, MBC is rare, and researchers pay less attention to it. Few studies, especially prospective ones, focus on MBC, resulting in a lack of objective and accurate clinical evidence for its treatment. Consequently, doctors often rely on treatment protocols established for female BC, adapting adjuvant chemotherapy indications and medications accordingly. Given that MBC patients are generally older, specific medications should be tailored to each patient's actual condition. Current literature on the prophylactic use of PEG‐rhG‐CSF post‐chemotherapy in BC patients indicates a paucity of studies addressing leukocytosis resembling leukemia reactions. Research has predominantly focused on female BC cohorts, with a notable absence of investigations into similar complications and therapeutic strategies among MBC patients post‐chemotherapy. Jiang et al. conducted an observational study involving 45 BC patients who received 6 mg of prophylactic PEG‐rhG‐CSF following chemotherapy. Among them, 17 patients exhibited leukocytosis resembling leukemia reactions, predominantly grade 1–2, demonstrating favorable tolerance [5]. Conversely, a MBC patient in this case report experienced significant leukocytosis post‐chemotherapy, peaking at 64.84 × 10^9^/L, accompanied by intermittent fever spikes reaching 42.5°C, indicative of poor tolerance to prophylactic 6 mg PEG‐rhG‐CSF. Hence, we present a case study documenting leukemia‐like reactions and associated complications following PEG‐rhG‐CSF administration in a MBC patient post‐chemotherapy.

The postoperative pathology of this patient revealed high‐risk factors such as grade III IDC with axillary lymph node metastasis, high expression of Ki‐67, and the presence of tumor thrombi in the vessels. According to NCCN guidelines, HR(+) and HER2(−) adjuvant chemotherapy is recommended as a dose‐dense AC‐T (dd AC‐T) regimen or AC‐T regimen [3]. High‐risk chemotherapy regimens for FN in BC include dd AC‐T, TAC, TCbH, and TC ± H regimens. Intermediate‐risk regimens include AC‐T, AC‐T ± HP, and TH [4]. The NCCN guidelines recommend primary prophylactic use of G‐CSF for patients receiving high‐risk FN chemotherapy regimens, regardless of whether the treatment's purpose is to cure, prolong survival, or improve disease‐related symptoms [3, 6]. For patients on intermediate‐risk regimens, prophylactic use of G‐CSF is recommended if they meet certain conditions such as age > 65, prior chemotherapy or radiotherapy, bone marrow involvement, recent surgery and/or open wounds, persistent neutropenia, renal or liver dysfunction, or HIV infection [4]. Prophylactic use of G‐CSF is not recommended for patients on low‐risk regimens unless they have specific risk factors [4].

Recombinant human granulocyte colony‐stimulating factor (rhG‐CSF) is effective in preventing CIN and FN [7]. The prophylactic use of G‐CSF during chemotherapy significantly reduces the incidence of FN [7]. PEG‐rhG‐CSF, the long‐acting form of rhG‐CSF, is a covalent combination of rhG‐CSF and polyethylene glycol (PEG) [8]. Studies have shown that PEG‐rhG‐CSF and rhG‐CSF are equally effective and safe in preventing CIN and FN induced by chemotherapy [7, 9]. Both drugs bind to stem cell receptors, promoting the proliferation, maturation, and release of neutrophils [7]. While rhG‐CSF has a short half‐life due to glomerular clearance and neutrophil‐mediated phagocytosis, PEG‐rhG‐CSF is primarily cleared by neutrophils and regulated by negative feedback from ANC, resulting in a longer half‐life [5, 10]. The incidence and persistence of grades 3 and 4 neutropenia are lower in patients treated with PEG‐rhG‐CSF compared to those treated with rhG‐CSF [8].

This patient was on a ddAC‐T regimen, a high‐risk chemotherapy regimen for FN. The NCCN guidelines recommend administering PEG‐rhG‐CSF once within 24–48 h at the end of each chemotherapy cycle, with at least a 12‐day interval before the next chemotherapy. The standard subcutaneous injection dose is 6 mg, with the option for individualized treatment based on the patient's body weight (100 μg/kg).

### 3.1 Diagnostic Workup and Approach to Management

The most common adverse reactions of PEG‐rhG‐CSF are mild to moderate bone pain, musculoskeletal pain, and allergic reactions [11]. Rare adverse reactions include leukocytosis resembling leukemia reaction, alveolar hemorrhage, hemoptysis, and acute respiratory distress syndrome (ARDS) [11]. The incidence of leukocytosis resembling leukemia reaction is reported to range from 28.6% to 37.8% [5], though its occurrence following chemotherapy in MBC patients remains rare. Leukocytosis resembling leukemia reaction is characterized by the leukocytes exceeding 50 × 10^9^/L, predominantly composed of neutrophils and/or immature cells. This condition is often accompanied by high fever, making it clinically challenging to differentiate from other potential causes of leukocytosis, such as bacterial infections (e.g., septicemia), malignant tumors (e.g., gastric cancer, lung cancer, renal cancer), and the convalescent stage of agranulocytosis. To accurately distinguish a neutrophilic leukemoid reaction from these other conditions, clinicians should follow a systematic diagnostic approach. Clinical evaluation is essential to assess the patient's symptoms, history of infections, and other relevant factors. A peripheral blood smear should be performed to look for signs of immature neutrophils, and further investigation should include blood cultures and other infection markers to rule out septicemia. When a malignant tumor is suspected, imaging studies (such as CT scans or PET scans) and tumor markers should be evaluated. Additionally, in cases where agranulocytosis is suspected, clinicians should consider the patient's recent chemotherapy history and look for signs of recovery from neutropenia, as well as reviewing bone marrow examination results to differentiate between marrow recovery and the onset of leukemia. A bone marrow aspiration may be necessary if the cause of leukocytosis remains unclear after these steps, especially in cases where atypical cells or blasts are observed on peripheral blood smear, or when bacterial cultures are negative and no other clear cause of neutrophilia is found. This helps to exclude hematological malignancies such as leukemia and other bone marrow disorders. The key to avoiding misdiagnosis is a comprehensive approach that includes thorough clinical assessment, appropriate laboratory testing, and when necessary, the use of invasive procedures such as bone marrow aspiration to confirm the diagnosis. Additionally, organizing temperature data and hematological analysis results into separate tables would greatly enhance the clarity and comprehension of the patient's clinical course, providing a clear visual representation of the timeline of symptoms and laboratory findings. This would assist clinicians in evaluating the progression of the leukocytosis and determining the most appropriate treatment and follow‐up strategies.

Leukocytosis resembling a leukemia‐like reaction generally does not require specific treatment beyond symptomatic management. Antipyretics, such as NSAIDs and dexamethasone, are effective in alleviating symptoms such as fever and bone pain. The leukocytes typically return to normal within 7 to 10 days, and the condition often resolves without the need for aggressive interventions. However, studies have shown that administering a half‐dose (3 mg) of PEG‐rhG‐CSF is associated with a higher incidence of FN compared to the full dose (6 mg) [12]. While dose reductions of PEG‐rhG‐CSF or rhG‐CSF are generally not recommended, they may be considered when the ANC exceeds 30 × 10^9^/L or the leukocyte count exceeds 50 × 10^9^/L prior to chemotherapy. In this case, adjusting the PEG‐rhG‐CSF dose to 3 mg, as done after the sixth chemotherapy cycle, effectively mitigated the leukocytosis resembling a leukemia‐like reaction and prevented further complications. This outcome emphasizes the importance of individualized dosing, particularly in patients exhibiting severe reactions. Clinicians should closely monitor these patients and adjust the PEG‐rhG‐CSF dose accordingly to manage the reaction while minimizing the risk of neutropenia and FN. Such dose adjustments could serve as a potential guideline for managing similar cases in the future, ensuring a balance between effective prophylaxis and the prevention of adverse effects.

### 3.2 Clinical Implications and Conclusion

**FIGURE 5:**
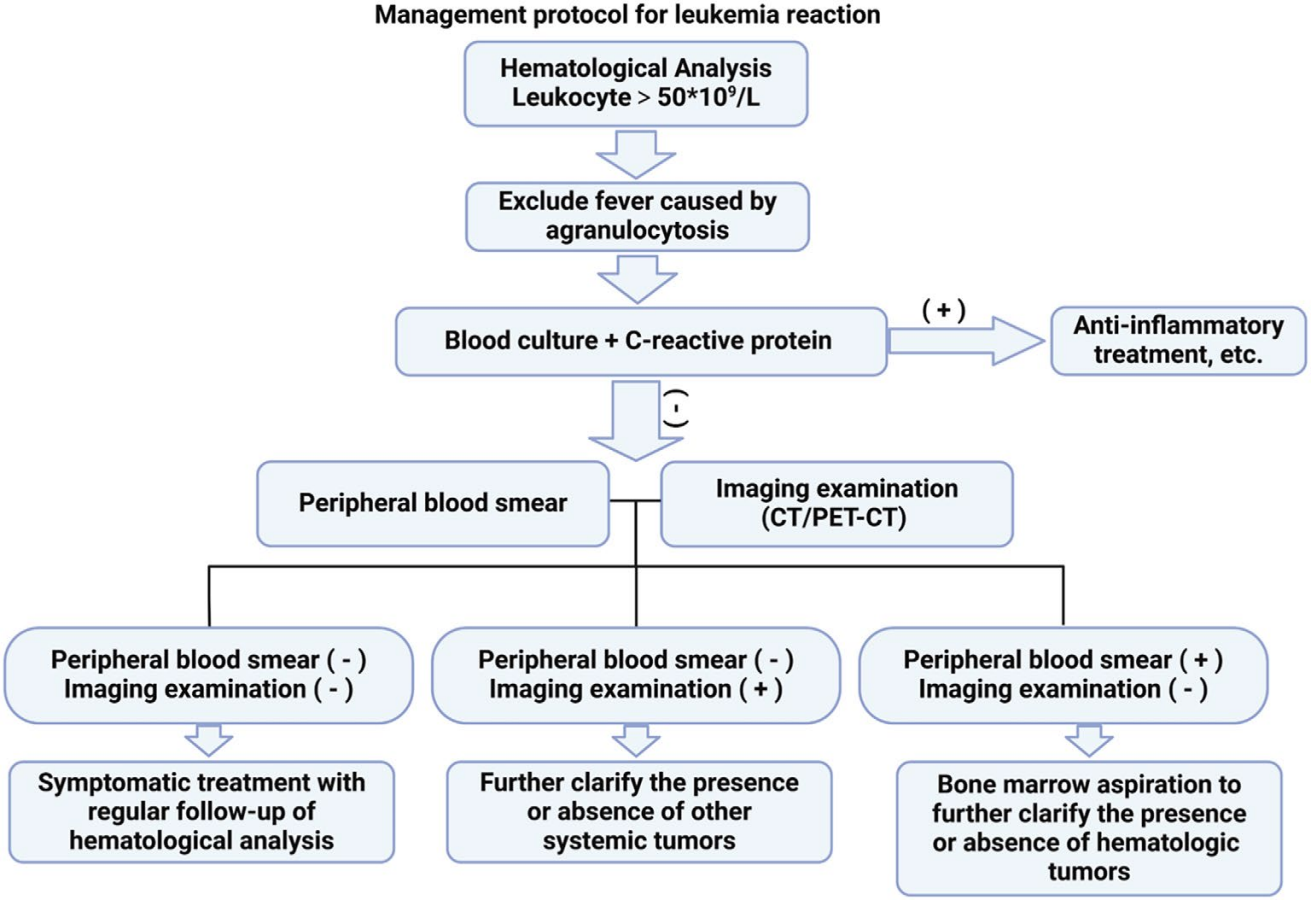
Leukemoid reaction process flow diagram.

There is limited literature on PEG‐rhG‐CSF‐induced leukocytosis resembling leukemia reaction in MBC patients after chemotherapy, which can lead to misdiagnosis, over‐examination, and overtreatment in clinical practice. The patient's case underscores the importance of careful monitoring and differential diagnosis, particularly in MBC patients, who are less studied in terms of chemotherapy‐induced complications. Despite the unusual and severe presentation in this case, with markedly elevated leukocytes and persistent fever, the leukocytosis resolved with symptomatic treatment and PEG‐rhG‐CSF dose adjustment. This case highlights that while PEG‐rhG‐CSF is effective in preventing CIN, clinicians should be aware of this rare adverse reaction, its diagnostic challenges, and the importance of tailored treatment approaches to avoid misdiagnosis and unnecessary interventions. The key takeaway from this case is the need for clinicians to maintain a high index of suspicion for PEG‐rhG‐CSF‐induced complications, particularly in MBC patients undergoing high‐risk chemotherapy regimens, and to employ a comprehensive diagnostic approach when managing such reactions (Figure 5).

Future studies are needed to further explore the optimal use of PEG‐rhG‐CSF in MBC patients and to establish more precise guidelines for managing chemotherapy‐induced complications in this population. Research focusing on personalized dosing strategies and the impact of PEG‐rhG‐CSF on MBC outcomes would help refine clinical practice and improve patient care.

## Author Contributions

Yanze Liu drafted the manuscript. Jiaqi Liu participated in the treatment and guided the writing of the paper.

## Conflicts of Interest

The authors declare no conflicts of interest.

## Supporting information


Data S1.

## Data Availability

The datasets analyzed during the current study are available from the corresponding author on reasonable request. The transparency in data reporting in accordance with journal requirements.
