# Supplementary material for: Male Breast Cancer Complicated With Leukocytosis Resembling Leukemia Reaction After Chemotherapy: A Case Report
Source: Cancer Rep (Hoboken). 2025 Jul 22;8(7):e70280. doi: 10.1002/cnr2.70280 (PMC12281595; doi:10.1002/cnr2.70280)
Supplement: Supplementary file 1 — Data S1. [file CNR2-8-e70280-s001.docx]

**Supplementary Table. Normal values of Leukocyte, ANC, CRP counts.**

| **Laboratory Tests** | **Normal values** |
| --- | --- |
| **Leukocytes** | **3.5 - 9.5** ×10⁹/L |
| **ANCs** | **1.4 - 7.2** ×10⁹/L |
| **CRP** | **0 - 5.00 mg/L** |

ANC, absolute neutrophil count; CRP, C-reactive protein.
